# Identification of a novel mutation in *ARSA* gene in three patients of an Iranian family with metachromatic leukodystrophy disorder

**DOI:** 10.1590/1678-4685-GMB-2016-0110

**Published:** 2017-11-06

**Authors:** Neda Golchin, Mohammadreza Hajjari, Reza Azizi Malamiri, Majid Aminzadeh, Javad Mohammadi-asl

**Affiliations:** 1 Noor Genetics Lab, Ahvaz, Iran; 2 Department of Genetics, Faculty of Science, Shahid Chamran University of Ahvaz, Ahvaz, Iran; 3 Department of Pediatric Neurology Golestan Medical, Educational and Research Center, Ahvaz Jundishapur University of Medical Sciences, Ahvaz, Iran; 4 Department of Pediatrics, Faculty of Medicine, Abuzar Children’s Hospital, Ahvaz Jundishapur University of Medical Sciences, Ahvaz, Iran; 5 Department of Medical Genetics, School of Medicine, Ahvaz Jundishapur University of Medical Sciences, Iran

**Keywords:** Metachromatic leukodystrophy disorder, *ARSA* gene, mutation, arylsulfatase A

## Abstract

Metachromatic leukodystrophy disorder (MLD) is an autosomal recessive and lysosomal storage disease. The disease is caused by the deficiency of the enzyme arylsulfatase A (ARSA) which is encoded by the *ARSA* gene. Different mutations have been reported in different populations. The present study was aimed to detect the mutation type of the *ARSA* gene in three relative Iranian patients. We found a novel homozygous missense mutation c.1070 G > T (p.Gly357Val) in exon 6 of these patients. The mutation was found to be reported for the first time in MLD patients. The data can update the mutation profile and contribute toward improved clinical management and counseling of MLD patients.

Metachromatic leukodystrophy (MLD, OMIM 250100) is a severe neurodegenerative disorder inherited in an autosomal recessive fashion. It is caused by deficiency of arylsulfatase A (ARSA, EC 3.1.6.8) protein. The deficiency results in the accumulation of glycolipid cerebroside sulfate in the myelin membranes of the central and peripheral nervous system (Lugowska *et al.*, 2014; Yaghootfam *et al.*, 2004).

Arylsulfatase A catalyzes the first step in the intralysosomal degradation of 3-O-sulfo-galactosylceramide (sulfatide). Sulfatide accumulation in myelin producing cells causes progressive destruction of white matter (leukodystrophy) throughout the nervous system. White matter damage causes progressive deterioration of intellectual functions and motor skills such as walking. These patients suffer almost exclusively from neurologic symptoms such as spatic tetraparesis, ataxia, optic atrophy, and dementia (Gieselmann *et al.*, 1991; Biffi *et al.*, 2008).

The prevalence of MLD varies among different population from 1 in 40,000 to 1 in 170,000 (Brimley *et al.*, 2013). Three clinical subtypes of MLD have been distinguished based on the age of onset: infantile (severe between 0-2 years), juvenile (3-16 years), and late onset (after sexual maturity). Approximately 50-60% of patients have the late infantile form, whereas 20-30% of the patients have the juvenile form, and 15-20% of patients have the adult form (Qu *et al.*, 1999; Liaw *et al.*, 2015).

Deficiency of arylsulfatase A is caused by mutations in *ARSA* gene. The *ARSA* gene (MIM #607574; GenBank accession number, NG_009260) is located on chromosome 22q13.33. This small gene (~3 kb) has eight exons and encodes a 509 amino-acid precursor (GenBank accession numbers, NM_000487.5 and NP_000478.3). The number of distinct *ARSA*–MLD allele types reported to date is about 200. Most of the mutations are missense type and so affect the encoded protein structure. However, it seems that the profile of mutations will be more updated in the future (Virgens *et al.*, 2015; Patil and Maegawa, 2013; Lugowska *et al.*, 2014).

Herein, we describe an Iranian affected family with a new mutation not reported before. The family has three patients with infantile MLD who were found to be homozygous for this mutation. Our results can update the mutation profile of this severe neurodegenerative disease.

Blood samples were collected from three affected and relative members of their family after obtaining informed consent. The participants underwent clinical examination. Arylsulfatase activity was measured by spectrophotometry (U2001, Hitachi, Japan). Genomic DNA was isolated from peripheral blood leukocytes using the QIAamp DNA Mini Kit (Qiagen, Hilden, Germany), according to the manufacturer’s instructions. All the eight coding exons and exon-intron boundaries of the *ARSA* gene were amplified using specific primers. PCR was performed using a thermal cycler (Model 2720; Applied Biosystems, Foster City, CA, USA). Direct sequencing was performed on an ABI Prism 3130XL Genetic Analyzer (Applied Biosystems) using the Big-Dye Terminator Cycle Sequencing Ready Reaction kit (Applied Biosystems, Foster City, CA, USA). Potential mutations were defined by exclusion from the Human Gene Mutation Database (http://www.hgmd.cf.ac.uk) and the previously reported mutations in PubMed (http://www.ncbi.nlm.nih.gov/PubMed/). We used computational tools including DUET, SDM, SNAP2, mutation tasting, and PolyPhen-2 to predict the potential impact of the mutation on protein function and structure. The ConSurf server, which identifies functional regions in the proteins, was also used to analyze the conservation of the desired amino acid.

The patients included three closely related children from three different parents in a large Iranian family (Figure 1). After clinical examination, it was found that EMG-NCV (Electromyogram and Nerve Conduction Velocity) in patients was abnormal, with a demyelinating sensorimotor neuropathy pattern causing severe motor and speech delay. In addition to clinical symptoms, brain MRI results were consistent with infantile MLD (Figure 2). Leukocyte ARSA A levels in the three patients ranged from 0.030 to 0.039 mu/mg protein, which is much lower than the normal range (0.375 – 1.815 mu/mg protein) (Table 1).

**Figure 1:**
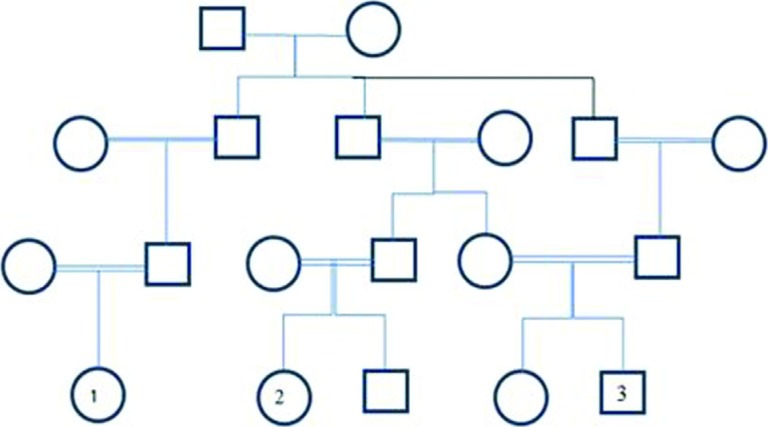
Pedigree of the family with MLD patients. The patients are shown in the fourth generation with the numbers 1, 2 and 3. The parents of patient 1 are paternal or maternal first cousins. Also, the parents of the patient 2 are first cousins once removed. It is of note that the information was provided by the parents of the patients. Genotypes of the patients and their parents are g.2207G>T (Hom) and g.2207G>T (Het) respectively.

**Figure 2:**
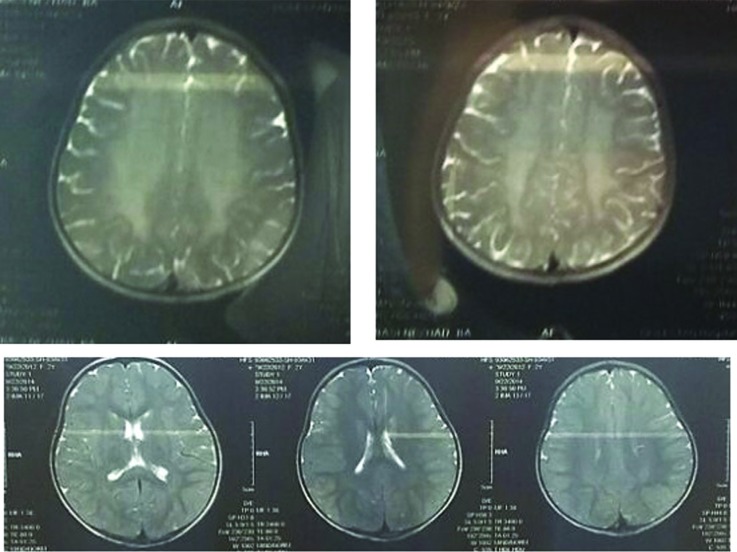
Magnetic resonance imaging T2W sequence in patients showing bilateral, diffuse confluent hyperintensities in white matter.

**Table 1:** Leukocyte ARSA A level and mutational analysis of ARSA

Case no.	Sex	Age	ARSA A (leukocytes) (mu/mg protein)	Amino acid change	Nucleotide change	Zygosity
**1**	Female	2yr 6mo	0.039(0.375-1.815)	Gly357Val	c.1070 G > T	Homo
**2**	Female	2yr 4mo	0.031(0.375-1.815)	Gly357Val	c.1070 G > T	Homo
**3**	Male	2yr	0.030(0.375-1.815)	Gly357Val	c.1070 G > T	Homo

Direct sequencing was done for all of the exons of the *ARSA* gene in the three patients. We found a novel homozygous missense mutation c.1070 G > T (p.Gly357Val) in exon 6 (Figure 3). Targeted mutation analysis of the patients parents showed that they were heterozygous for this allele at the *ARSA* locus. A summary of the results of *ARSA*A level and mutational analysis is given in Table 1.

**Figure 3:**
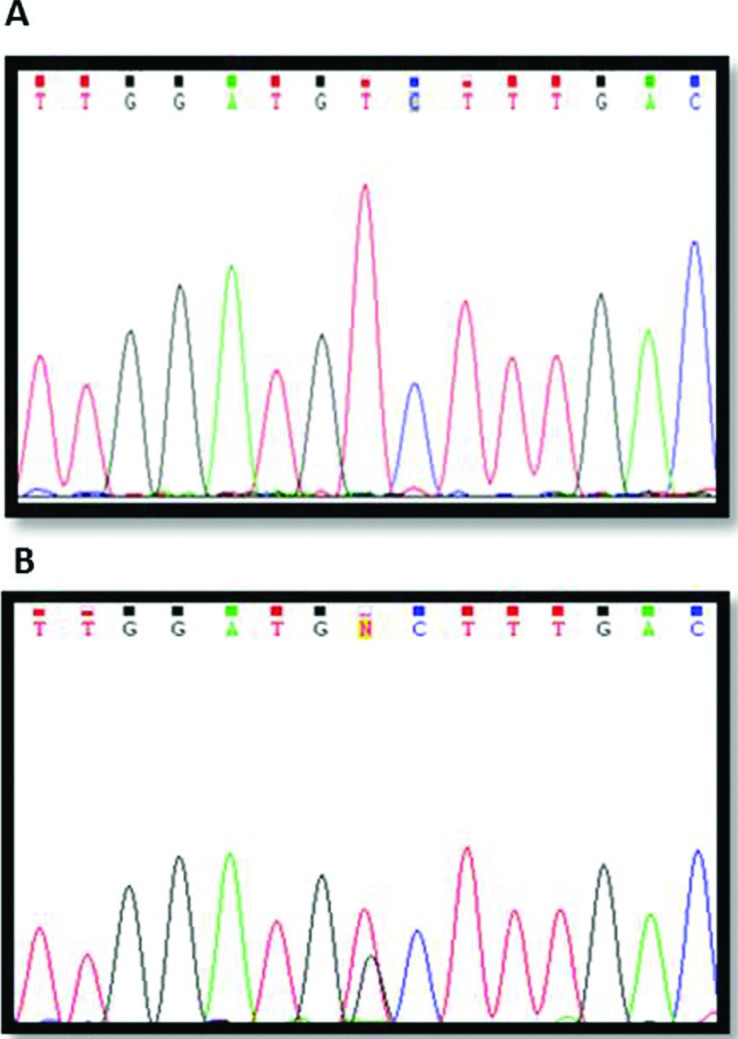
Mutational analysis of the arylsulfatase A (*ARSA*) gene in the Iranian family with metachromatic leukodystrophy. Direct sequencing of the *ARSA* gene shows a homozygous G to T transition (c.1070; Gly357Val) in patients (no. 1, 2, 3 in pediatric) (A), and a heterozygous G to T transition in their parents (B).

All of the bioinformatics analyses confirmed that the aforementioned change is a pathogenic mutation. The programs showed that the mutation can cause protein malfunction and disease. The ConSurf server also showed the conservation of the involved amino acid in different mammals.

Metachromatic leukodystrophy is an autosomal recessive disorder and lysosomal storage disease caused by ARSA deficiency. The disease results in demyelination in the central and peripheral nervous system (Lugowska *et al.*, 2011; Cesani *et al.*, 2009). Although various mutations in the *ARSA* gene have been reported, the recent studies show that there are still mutations that remain to be discovered. Finding these mutations can help genetic counseling of families with MLD patients.

In the current study, we described a new point mutation in exon 6 of the *ARSA* gene in three Iranian patients with infantile type MLD. The three late-infantile MLD patients, who showed severe clinical characteristics, were the children of three consanguineous marriages in a large family. This mutation results in an amino-acid substitution of glycine (GGC) with valine (GTC) at codon 357 of the mRNA coding *ARSA* protein.

Different bioinformatics algorithms showed that the mutation is expected to be pathogenic. The DUET program, which is a server for predicting the effects of mutations on protein stability using two complementary approaches (mCSM and SDM) in a consensus prediction, showed that the mutation may destabilize the protein. In addition to the prediction of protein stability, the mutation testing program also reported that splice sites might be changed due to the gain of a new potential donor site.

Different mutations have been identified in the *ARSA* gene, including substitution and small deletions/insertions (Stoeck *et al.*, 2016). Common mutations such as c.459+1 G > A, c.1204+1 G > A, p.Pro426Leu and p.Ile179Ser exist in certain populations including western European populations (Lugowska *et al.*, 2005). Most of the reported mutations in the *ARSA* gene are missense type. Three mutations including p.Gly99Asp, p.Gly245Arg and p.Thr409Ile are also the most common types in Japanese MLD patients (Hayashi *et al.*, 2011; Cesani *et al.*, 2016). However, the most common mutation is the splice donor site mutation of the exon2/intron2 border, IVS459+1G > A, which is associated with the late infantile clinical form of MLD. Although several missense mutations have been identified, few of them have been biochemically characterized. Patients homozygous for alleles not producing functional ARSA always develop the most severe late-infantile form of MLD (Berger *et al.*, 1997, Hayashi *et al.*, 2011; Kang *et al.*, 2010).

Since it seems that a broad spectrum of mutations is associated with MLD, the diagnostic strategies should detect both common and rare MLD alleles. To our knowledge, our study is the first report for this specific mutation in MLD patients. The substitution of glycine with valine is clearly non-conservative and seems to have an effect on protein stability. Moreover, the conservation of glycine in different mammals indicates its importance in the structure and function of ARSA. However, future studies of transfected cells with the new mutation are needed to investigate its structural impact on arylsulfatase A.
